# More or less—On the influence of labelling strategies to infer cell population dynamics

**DOI:** 10.1371/journal.pone.0185523

**Published:** 2017-10-18

**Authors:** Michael Gabel, Roland R. Regoes, Frederik Graw

**Affiliations:** 1 Center for Modelling and Simulation in the Biosciences, BioQuant-Center, Heidelberg University, 69120 Heidelberg, Germany; 2 Institute for Integrative Biology, ETH Zurich, CH-8092 Zurich, Switzerland; University of Glasgow, UNITED KINGDOM

## Abstract

The adoptive transfer of labelled cell populations has been an essential tool to determine and quantify cellular dynamics. The experimental methods to label and track cells over time range from fluorescent dyes over congenic markers towards single-cell labelling techniques, such as genetic barcodes. While these methods have been widely used to quantify cell differentiation and division dynamics, the extent to which the applied labelling strategy actually affects the quantification of the dynamics has not been determined so far. This is especially important in situations where measurements can only be obtained at a single time point, as e.g. due to organ harvest. To this end, we studied the appropriateness of various labelling strategies as characterised by the number of different labels and the initial number of cells per label to quantify cellular dynamics. We simulated adoptive transfer experiments in systems of various complexity that assumed either homoeostatic cellular turnover or cell expansion dynamics involving various steps of cell differentiation and proliferation. Re-sampling cells at a single time point, we determined the ability of different labelling strategies to recover the underlying kinetics. Our results indicate that cell transition and expansion rates are differently affected by experimental shortcomings, such as loss of cells during transfer or sampling, dependent on the labelling strategy used. Furthermore, uniformly distributed labels in the transferred population generally lead to more robust and less biased results than non-equal label sizes. In addition, our analysis indicates that certain labelling approaches incorporate a systematic bias for the identification of complex cell expansion dynamics.

## Introduction

The ability to distinguish cells and organisms by certain markers and labels has been an indispensable asset in many biological experiments addressing population dynamics and development. For example, tracking differently labelled cells not only allows the identification of lineage pathways [1], but also the observation of dynamical changes in cell populations over time [2]. The application of labels also helps to determine the migration dynamics of cells between organs [3], or the colonisation dynamics of specific tissues by bacteria [4, 5]. In addition, the information obtained by labelling can be used to quantify cellular turnover, such as cell activation, proliferation and differentiation dynamics [6].

For cells, there exists a large variety of experimental techniques to label and track individual populations. Besides the application of markers that are taken up during cell proliferation, such as BrdU [7, 8], deuterated glucose and heavy water [9–11], this especially concerns techniques that involve the adoptive transfer of pre-labelled cell populations. Staining cells by the fluorescent dye CFSE [12, 13] has been used extensively to infer cellular turnover and proliferation dynamics (reviewed in [6]). More fine-grained approaches that involve several different markers—e.g. by transferring cell populations bearing congenic markers [14–16] or by using naturally diverse markers, such as T cell receptor sequences [17–20]—allow to distinguish the dynamics of individual subpopulations in more detail. Finally, artificially labelling cells by unique, inheritable genetic barcodes makes it possible to follow cellular dynamics on a single cell level [21]. By this, one is able to address cell heterogeneity and to identify individual cell differentiation pathways [2, 21–23].

The adoptive transfer of labelled cells is particularly useful, if the experimental conditions prevent sampling at different times. When organs or cell cultures need to be harvested, individual measurements can only be obtained at one particular time point. In these cases, the intra-individual variability in the population dynamics of each label can provide enough information to estimate cellular turnover. Interestingly, it is also possible to quantify interacting dynamics, such as entangled migration and proliferation dynamics, even if measurements are only obtained from one of the involved compartments [4]. Thus, using multiple labels can compensate for both the lack of time-resolved data and compartments that cannot be measured.

Several different labelling strategies have been used to analyse population dynamics given these experimental limitations. These approaches differed in the number of labels and the size of each label within the transferred population [2, 4, 16]. However, it has not been determined so far if these labelling strategies allow to reliably infer the assumed dynamics, and how these different approaches influence the quantification of the kinetics: does the estimation of a cell proliferation rate benefit from a high or a small number of cells per label? To what extent would parameter estimation be improved if more labels are used? And how does the time point of sampling affect parameter identification? The impact of a labelling strategy on parameter identification needs to be evaluated in order to determine the reliability of obtained parameter estimates.

**Fig 1:**
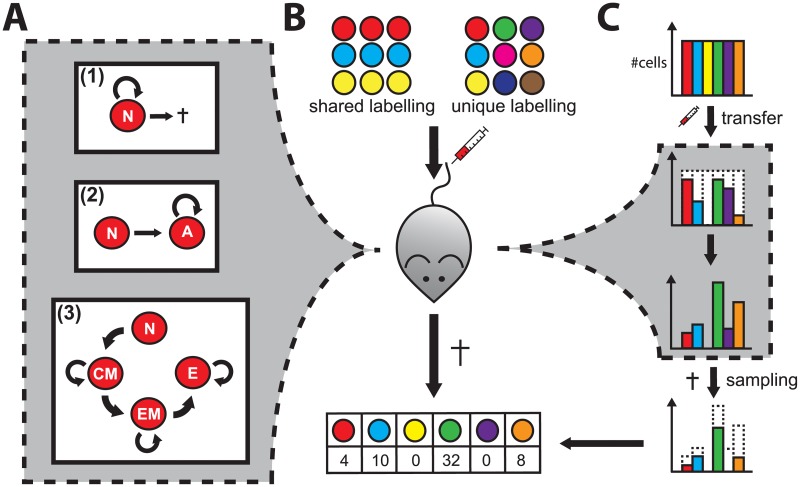
Population dynamics, experimental setups and technical shortcomings. (**A**) Schematic of different models with increasing complexity describing cellular turnover: (**1**) *Homoeostatic turnover*: Naïve cells proliferate only to compensate cell death, therefore maintaining a stable number of cells. (**2**) *Simple expansion dynamics*: By encountering their respective antigen naïve cells are activated and start to proliferate. (**3**) *Complex expansion dynamics*: In comparison to (2), we consider several steps of cell differentiation and proliferation. Upon activation, naïve cells differentiate into central memory precursor (CM) and subsequently into effector memory precursor cells (EM), and finally effector cells (E). For simplicity, net-proliferation rates combining cell proliferation and death are considered at this point [16]. (**B**) A labelling strategy using inheritable labels is defined by the number of different labels and the label size, i.e. the number of cells per label. The depicted labelling strategies show a shared and a unique labelling approach. After transfer into a host, these cells are thought to follow one of the three cellular dynamics. At a specific time, cells are sampled and used for evaluation. Data are gathered in the form of *count* data measuring the number of cells of a specific label within the sampled population. (**C**) Potential experimental shortcomings: Cells can be lost during transfer and/or sampling.

To this end, we studied the appropriateness of different labelling strategies to quantify cellular dynamics. Here, we focus on labelling approaches based on inheritable and stable markers, such as congenic markers or genetic barcodes. We considered cellular systems of various complexity that assumed either homoeostatic turnover, as e.g. for naïve T cells, or cell expansion dynamics involving various steps of cell differentiation and proliferation (Fig 1A). We then simulated adoptive transfer experiments varying the composition of the labelled cell population (Fig 1B). Data sampled at a single time point were used to quantify the underlying kinetics and to evaluate the impact of the labelling strategy on parameter estimation. In addition, we analysed how experimental shortcomings, such as incomplete transfer or sampling of the labelled cell population (Fig 1C), affected the results.

Our results show that labelling strategies and the experimental limitations affect parameter estimates in multiple ways: The appropriateness of a labelling approach depends on the underlying cellular system, but also on the type of parameter that is to be identified. Labelling strategies might be biased to favour the identification of certain types of cellular characteristics, e.g. proliferation rates, with some approaches being more robust than others with respect to loss of cells during transfer or sampling.

In general, our findings argue for the use of multiple labels with an intermediate number of cells per label to reliably infer cellular transition and expansion dynamics. Furthermore, they also suggest the use of simulations to determine a-priori the appropriateness and limitations of the experimentally used labelling strategy, or to later validate obtained parameter estimates.

## Materials and methods

### The mathematical models of cellular dynamics

We distinguish three scenarios of cellular dynamics that consider different levels of complexity (see Fig 1A). These scenarios are described as follows.

#### (1) Homoeostatic turnover

Under homoeostatic conditions, a cell population is considered to be in equilibrium, meaning the total number of cells is assumed to be constant over time. However, the cell population is usually not static as cells constantly die and are replaced. Examples of homoeostatic turnover among immune cells are the dynamics of naïve T cells before antigen encounter, or the pool of memory T cells that is maintained after an infection [24]. In our model, we assume that a cell population, here termed naïve cells, *N*, proliferates with rate *ρ* and dies with rate *δ*. The dynamics are described by the following differential equation:

$$\begin{array}{c} \frac{dN}{dt}=\left(\rho -\delta \right)N. \end{array}$$ (1)

To ensure homoeostatic turnover, *ρ* = *δ*. In the following, we set *ρ* = *δ* = 0.5 d^−1^ to allow for reasonable simulation times. This is done without loss of generality as the timescale of the simulations can be rescaled to allow interpretation for much lower turnover rates, as e.g. observed for naïve T cells [6] (see also S1 Fig).

#### (2) Simple expansion dynamics

Another dynamics is the activation and subsequent proliferation of cells (Fig 1A). After encountering their cognate antigen, naïve T cells are activated and start to fight effectively against the invading pathogen by massively expanding in numbers and simultaneously differentiating into effective subpopulations [16]. To model a simple expansion dynamics, we distinguish between naïve, *N*, and activated cells, *A* [25]. Naïve cells are activated with rate *μ*, and activated cells start to proliferate with rate *ρ*. The dynamics of this model can be described by the following system of ordinary differential equations:

$$\begin{array}{c} \begin{array}{cc} \frac{dN}{dt} & =-\mu N\\ \frac{dA}{dt} & =\mu N+\rho A \end{array} \end{array}$$ (2)

For simplicity, cell death of both naïve and activated cells is neglected in this model, as we are mainly interested in the net-expansion rates.

#### (3) Complex expansion dynamics

In a third step, we extended the simple expansion model by additionally accounting for heterogeneous subpopulations among the activated cells. As for example for T cells, several functionally diverse subsets are distinguished that indicate different steps of cell differentiation [2, 26]. Each of these subsets is assumed to follow individual proliferation and differentiation dynamics. Following the study by Buchholz et al. [16], we distinguish between central memory precursor (CM), effector memory precursor (EM) and effector cells (E). The relation between these compartments is assumed to follow a linear differentiation pathway as depicted in Fig 1A and is defined by the following system of ordinary differential equations:

$$\begin{array}{c} \begin{array}{cc} \frac{dN}{dt} & =-\mu_{N}N\\ \frac{dCM}{dt} & =\mu_{N}N+\left(\rho_{\text{CM}}-\mu_{\text{CM}}\right)CM\\ \frac{dEM}{dt} & =\mu_{\text{CM}}CM+\left(\rho_{\text{EM}}-\mu_{\text{EM}}\right)EM\\ \frac{dE}{dt} & =\mu_{\text{EM}}EM+\rho_{E}E, \end{array} \end{array}$$ (3)

where *μ*_*x*_ and *ρ*_*x*_ describe the differentiation and proliferation rates, respectively of the corresponding compartments. We used estimates derived from Buchholz et al. [16] to parametrise the model; the respective values are given as *μ*_N_ = 2.2 d^−1^, *μ*_CM_ = 0.2 d^−1^, *μ*_EM_ = 0.04 d^−1^, *ρ*_CM_ = 0.85 d^−1^, *ρ*_EM_ = 1.42 d^−1^ and *ρ*_E_ = 1.6 d^−1^.

### Simulating labelling experiments

To simulate experimental data, we performed stochastic simulations of the systems defined by Eqs (1)–(3) based on the Gillespie algorithm [27]. Simulations were carried out in the R-language of statistical computing using the package *adaptivetau* [28]. Each simulation starts with a specified number of labelled naïve cells at time *t* = 0. These cells then proliferate, differentiate or die stochastically, according to the underlying model. We assume inheritable markers, meaning that the label of each individual cell is retained during activation or differentiation, and it is passed onto every daughter cell while proliferating. At a specified sampling time *T* > 0 the system is stopped and the number of cells per label in each cellular subset is assessed.

In addition to the model parameters characterising the cellular dynamics, each simulation depends on the following experimental parameters: The sampling time, *T*, at which cells are sampled, and the labelling strategy, which is defined by the number of different labels, *L*, and the label size *M*, i.e. the number of cells per label in the initial cell population. Unless stated otherwise, we assume uniformly distributed labelling strategies, i.e. every label has initially the same number of cells.

To account for possible loss of cells during transfer (Fig 1C), the fraction of cells that is assumed to pass the transfer is sampled randomly from the initial cell population. This sampled transfer fraction is then used as an initial condition for the model systems. Similarly, by randomly sampling a predefined fraction of cells from the stochastically generated simulation output, we account for incomplete sampling that might occur during experiments. The sampled cell population is then used to estimate the parameters of the underlying system.

### Parameter estimation

Parameter estimates for the rates describing cell activation, proliferation and differentiation are obtained by fitting the predicted summary statistics for each cell population to the sampled count data, which provide the absolute number of cells for each label. Each sample is evaluated individually. The considered summary statistics include the expected mean, the coefficients of variation (CV) and, if applicable, the correlation coefficients (CC). The predicted summary statistics are obtained by solving the corresponding master equations of the systems (Eqs (1)–(3)) (see S1 Appendix for a detailed description of the calculations). Fitting is then performed based on *χ*^2^-minimisation using the optim-function in the R-language of statistical computing [28].

Confidence intervals for parameter estimates are obtained by bootstrapping the data using the built-in R-package boot. These intervals are calculated based on Efron’s non-parametric and accelerated bootstrap (BC*a*) method [29] with 999 repeats and a significance level of *α* = 0.05.

To allow for comparison with the original approach by Buchholz et al. [16], the compartment of naïve cells, *N*, was not considered when fitting both the simple and the complex expansion model.

### Evaluating the quality of parameter estimates

The appropriateness of different labelling strategies to retrieve the underlying cellular dynamics is determined by different quantities [30]. These quantities characterise the robustness of parameter estimates and their deviation from the true parameter.

#### Bias

The bias indicates on an absolute scale how much the average parameter estimate deviates from the true value.

In mathematical terms, if ${\hat{\theta }}_{i}$, *i* = 1, …, *m* are estimates for the true parameter *θ* with $\hat{\theta }:=\frac{1}{m}\sum_{i=1}^{m}{\hat{\theta }}_{i}$ defining the empirical mean, the bias is calculated by

$$\begin{array}{c} \text{Bias}\;≔\;\hat{\theta }-\theta . \end{array}$$ (4)

#### Percentage bias

The percentage bias determines on a relative scale how much the average estimate differs from the true value. This allows the simultaneous comparison of estimates for several parameters of different scales.

The percentage bias is defined by

$$\begin{array}{c} \text{pBias}\;≔\;\frac{\hat{\theta }-\theta }{\theta }. \end{array}$$ (5)

#### Mean confidence interval length (MCIL)

The MCIL serves as a measure of uncertainty for the parameter estimate. If CI_*i*_ = [*a*_*i*_, *b*_*i*_] is the estimated confidence interval for parameter *θ* in run *i*, with *l*(CI_*i*_) = *b*_*i*_ − *a*_*i*_ defining the length of the confidence interval, then the mean confidence interval length is calculated by

$$\begin{array}{c} \text{MCIL}\;≔\;\frac{1}{m}\sum_{i=1}^{m}\;l\left({\text{CI}}_{i}\right). \end{array}$$ (6)

Here, *m* denotes the total number of individual runs performed. In some cases the MCIL cannot be calculated (e.g. due to an unlimited confidence interval of at least one of the confidence intervals used for calculation). This is indicated in the corresponding plots by a grey coloured box for the respective parameter combination.

#### False coverage rate (FCR)

The false coverage rate is defined as the fraction of simulation runs in which the estimated confidence interval does not contain the predefined rate.

## Results

### The influence of transfer loss on parameter estimation

During adoptive transfer of cells into a living host, it is unlikely that all cells will survive the transit. Common obstacles include experimental limitations, such as imperfect injections to the target tissue, or host-induced rejection of cells, e.g. when using congenic markers [31]. Therefore, one would expect that only a fraction of the original labelled cell population enters the system and can be recovered later. As we show below, neglecting this *transfer fraction* when evaluating sampled data can strongly impact parameter estimates of the cellular dynamics.

**Fig 2:**
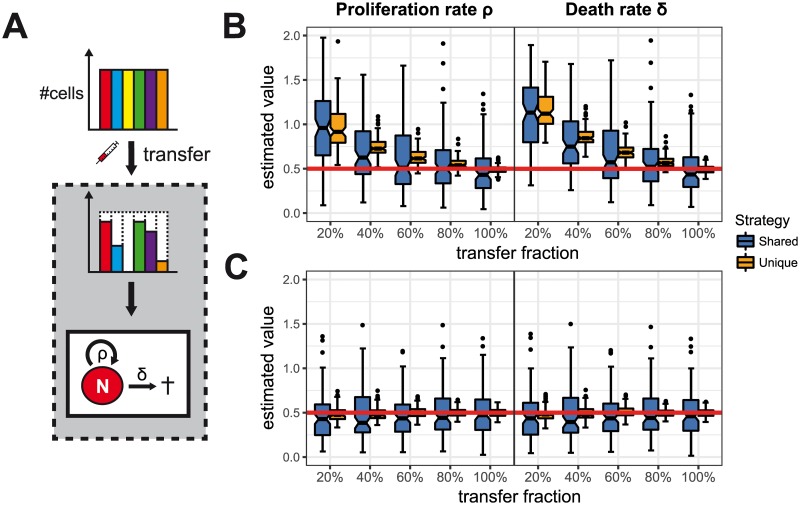
The influence of incomplete transfer on estimating homoeostatic cell turnover. (**A**) Only a fraction of the initially labelled cell population might survive the transfer and follows homoeostatic turnover where cells proliferate with rate *ρ* and die at rate *δ*. (**B**) Panels show the distribution of estimates for the proliferation rate, *ρ* (left), and the death rate, *δ* (right), for different fractions of cells surviving the transfer. Parameter estimates for two labelling strategies with *N* = 800 cells initially using either shared (*L* = 8, *M* = 100, *blue*) or unique labelling (*L* = 800, *M* = 1, *orange*) are shown. The estimation procedure did not account for the transfer loss. (**C**) Prior knowledge on the transfer loss improves parameter estimates even if only small fractions of cells survive the transfer. Each boxplot is based on the results of 100 individual stochastic simulations with *ρ* = *δ* = 0.5 d^−1^ and cells being sampled 8 days after transfer. Red lines indicate the true parameter values.

Assuming homoeostatic cell turnover where cells proliferate and die at similar rates, we tested the ability of two different labelling strategies to infer the underlying kinetics in case of incomplete transfer (Fig 2A). Both labelling strategies involve the adoptive transfer of *N* = 800 cells that are labelled according to a unique (*L* = 800 labels with *M* = 1 cell each) or a shared labelling approach (*L* = 8, *M* = 100); both of which have been successfully used in experiments [2, 16]. Cells that survive the transfer and undergo stochastic homoeostatic turnover are sampled at a single time point and used for parameter estimation.

Incomplete transfer results in an overestimation of both the proliferation and the death rate in either of the two labelling strategies (Fig 2B). In addition, the parameter estimates indicate an exponential decay of cells rather than a homoeostatic turnover as the death rate *δ* is always estimated to be higher than the corresponding proliferation rate *ρ*. This is due to the fact that the system has to compensate for the smaller number of cells that are recovered compared to the number of labelled cells in the inoculum. A higher transfer loss also results in a larger variation of the parameter estimates whereby a unique labelling approach allows more robust estimation.

If prior knowledge on the transfer fraction can be obtained, as for example by additional experiments [16], it is possible to adjust the estimation procedure and to obtain appropriate parameter estimates for the homoeostatic turnover (see Fig 2C and S1 Appendix). This works reliably for both labelling strategies even if large fractions of cells are lost during transfer.

**Fig 3:**
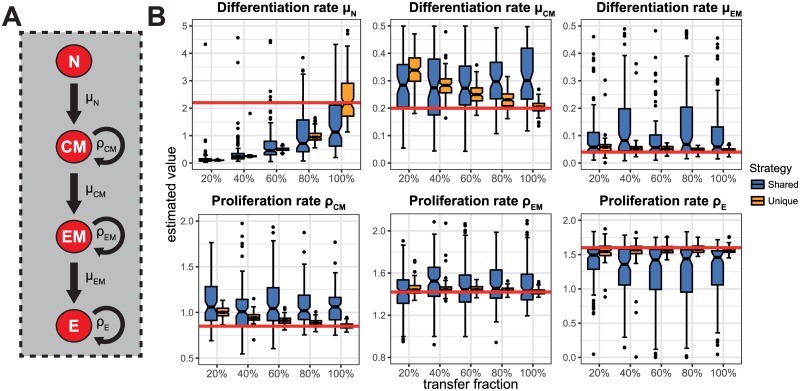
The influence of incomplete transfer on estimating complex cell differentiation and expansion dynamics. (**A**) Schematic of a linear pathway for cell differentiation and proliferation as assumed for the complex expansion model [16]. Naïve cells (N) turn into central memory precursor cells (CM), which subsequently turn into effector memory precursor (EM) and effector (E) cells. Cells differentiate and proliferate according to the corresponding rates *μ* and *ρ*, respectively. (**B**) Panels show the distribution of estimates for the differentiation (upper row) and proliferation rates (lower row) for the different cellular subsets for various fractions of cells surviving the transfer. The estimation procedure did not account for the transfer loss. Parameter estimates for two labelling strategies with *N* = 800 cells initially using either shared (*L* = 8, *M* = 100, *blue*) or unique labelling (*L* = 800, *M* = 1, *orange*) are shown. Every boxplot is based on the results of 100 individual stochastic simulations. Differentiation and proliferation rates are defined as *μ*_N_ = 2.2 d^−1^, *μ*_CM_ = 0.2 d^−1^, *μ*_EM_ = 0.04 d^−1^, *ρ*_CM_ = 0.85 d^−1^, *ρ*_EM_ = 1.42 d^−1^ and *ρ*_E_ = 1.6 d^−1^ [16]. Red lines indicate the true parameter values.

However, in more complex scenarios of cell expansion dynamics, even full knowledge on the transfer fraction might not be sufficient to correctly quantify the underlying dynamics. Buchholz et al. [16] studied the proliferation and differentiation dynamics of T cells and identified a linear differentiation pathway with naïve (N) cells differentiating into central memory (CM) and effector memory precursor cells (EM), and further into effector cells (E) (Fig 3A). Using a shared labelling strategy adapted from their experiment, we find that all parameter estimates besides the naïve differentiation rate *μ*_*N*_ seem unaffected by a loss of cells during transfer (Fig 3B). Accounting for the transfer fraction in the estimation procedure leads to more robust but not necessarily correct estimates (S2 Fig). Even if no cells are lost during transfer, the proliferation and differentiation rates associated with the naïve and central memory compartment, i.e. *μ*_*N*_, *μ*_*CM*_ and *ρ*_*CM*_, are under- and overestimated with a relative bias of 0.5 and 1.5, respectively (Fig 3B). In contrast, the unique labelling approach allows identification of the underlying kinetics for all cellular subsets if all cells survive the transfer, or if the effective transfer fraction is known and accounted for (Fig 3B, S2 Fig). If the transfer fraction is not known, this labelling approach also leads to biased estimates of the proliferation and differentiation rates *μ*_*N*_, *μ*_*CM*_ and *ρ*_*CM*_, but does not affect the estimates of the remaining parameters. As in the homoeostatic scenario, the larger number of labels of the unique labelling strategy leads to less variation in the parameter estimates compared to the shared labelling approach.

In summary, our results show that incomplete transfer mainly affects the quantification of cellular kinetics in early compartments while the estimation of later differentiation and expansion steps is not affected.

### The influence of incomplete sampling on parameter estimation

Sampling cells from the host system represents another source of error. Most likely only a fraction of the labelled cell population can be recovered as cells migrate into different tissues or are lost during circulation [32]. In addition, pre-treatment of harvested tissue for experimental measurements can lead to additional loss of cells [33]. To determine the impact of incomplete sampling on the quantification of cellular kinetics we repeated our analysis but only considered a fraction of the cell population at the time point of sampling in the estimation procedure. For simplicity, we assumed that all cells survived the adoptive transfer.

**Fig 4:**
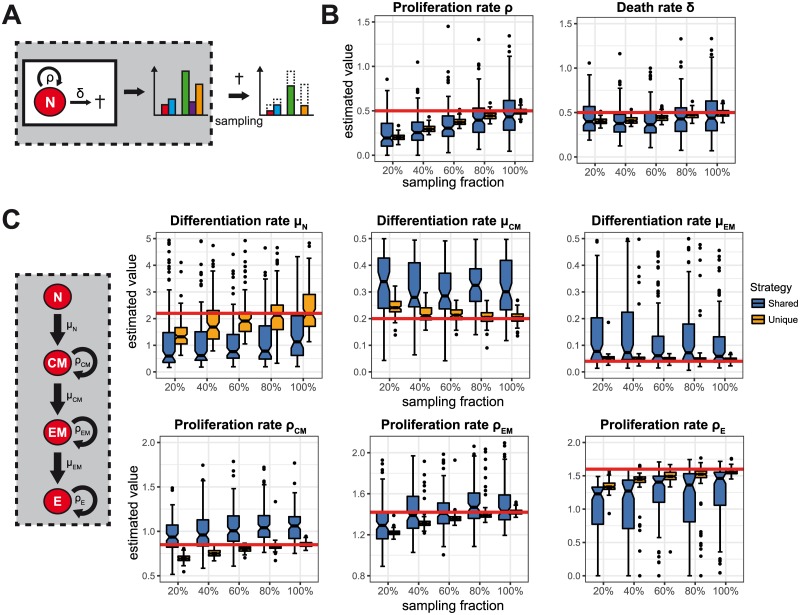
The influence of incomplete sampling on parameter estimation. (**A**) Schematic depicting the problem of incomplete sampling: Only a fraction of the labelled cells is sampled and can be used for analysis. (**B**) Panels show the distribution of the estimated proliferation, *ρ*, and death rate, *δ*, for the homoeostatic system shown in (**A**) using the shared (*blue*) and unique (*orange*) labelling approach given different sampling fractions. (**C**) The distribution of the estimated parameters for the complex expansion dynamics analogous to (**B**). Every boxplot is based on the results of 100 individual stochastic simulations. Parameters used to simulate the dynamics and the time point of sampling are defined as before. Red lines indicate the true parameter values.

Given homoeostatic cell turnover, not accounting for incomplete sampling results in an underestimation of both the proliferation, *ρ*, and the death rate, *δ* (Fig 4B). This bias decreases with increasing sampling fractions. However, in comparison to a scenario with incomplete transfer, the relative bias of the death rate is on average substantially smaller than the relative bias of the proliferation rate (compare Fig 2B). These observations can be seen for both labelling strategies used.

In contrast, more diverse effects are observed when quantifying the cellular dynamics in the complex expansion model (Fig 4C). Incomplete sampling seems to affect the two types of rates, i.e. differentiation and proliferation rates, differently: While estimates of the differentiation rates are not affected by different sampling fractions, proliferation rates are generally underestimated. This trend is especially visible for the unique labelling approach, while shared labelling is less affected by incomplete sampling (Fig 4C). However, as already seen for incomplete transfer, the shared labelling approach leads to a biased estimation of the cellular dynamics, especially for non-intermediate compartments, e.g. N, CM and E (Fig 4C).

Similar to the shortcoming of incomplete transfer, incomplete sampling can be addressed in the estimation procedure by rescaling the measured cell numbers by the sampled fraction (see S3 Fig). However, it might be experimentally difficult to obtain an estimate for this fraction.

Thus, while incomplete transfer especially affects the quantification of transition rates, incomplete sampling particularly leads to underestimation of the proliferation rates.

### The influence of labelling strategies on parameter identification

Our previous analyses indicate that the composition of the labelled cell population affects parameter estimates. The unique labelling approach leads to more robust and less biased estimates than the shared labelling strategy (Figs 2–4). This increased robustness is expected, as the unique labelling strategy provides up to 800 individual measurements, i.e. one for each label. This is 100-fold the number we obtain when using the shared labelling approach. However, the latter strategy might still comprise useful aspects, because less labels are lost by stochastic effects or during sampling due to the larger number of cells per label. In addition, larger population sizes usually allow more robust experimental measurements.

In order to investigate the qualitative influence of different labelling strategies on the quantification of cellular dynamics, we studied a system of simple cell expansion in which transferred cells, *N*, are activated with an activation rate *μ* and activated cells, *A*, proliferate with rate *ρ* (Fig 1A) [25]. Here, we analysed the impact of different factors on the ability to infer the cellular kinetics. This included (i) the actual labelling strategy for the transferred cell population characterised by the number of labels, *L*, and the number of cells per label, *M*, (ii) the sampling time, *T*, and (iii) the activation, *μ*, and proliferation rate, *ρ* that determine the cellular dynamics. To focus on the impact of each individual factor, we always assumed complete transfer and sampling.

#### Influence of the labelling strategy

By varying the number of labels, *L*, from 2 to 50 and the number of cells per label, *M*, from 1 to 50, we assessed the influence of a total of 2450 different labelling strategies on their ability to quantify the cellular turnover.

**Fig 5:**
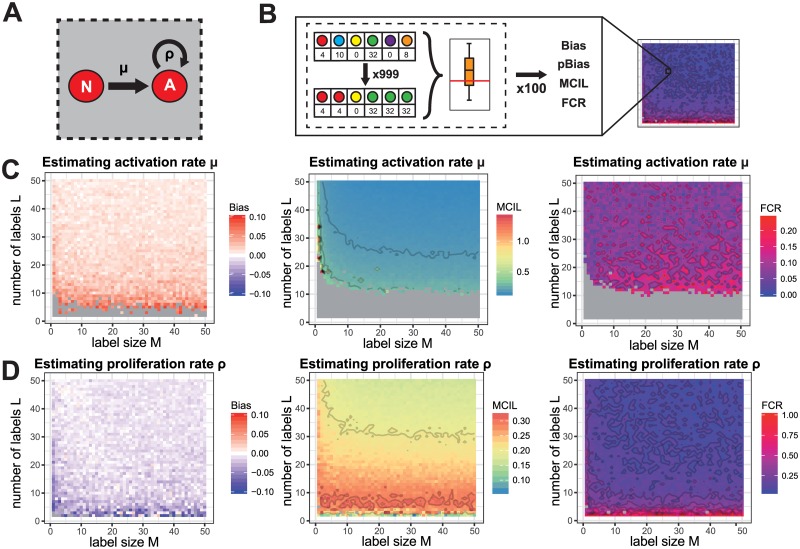
The influence of the labelling strategy on parameter estimates. (**A**) Schematic depicting the dynamics of the simple expansion model: Cells are activated with rate *μ* and activated cells proliferate with rate *ρ*. (**B**) Calculation of heatmaps: Each labelling strategy is used to generate 100 stochastically simulated data samples. Each data sample is then bootstrapped with 999 repeats (see Materials and Methods) to calculate the corresponding distribution of parameter estimates and the respective confidence interval. Combining these results allows the calculation of the depicted statistical quantities for the corresponding parameter combination. (**C-D**) The bias, the mean confidence interval length and the false coverage rate for the estimation of the activation rate, *μ* (**C**), and the proliferation rate, *ρ* (**D**), assuming a system of simple expansion dynamics. The estimation for each parameter combination is based on 100 independent stochastic simulations. Parameters not varied are fixed to *μ* = 0.3, *ρ* = 0.3 and cells were sampled at *T* = 3. Grey colour indicates values being above or below the shown range (Bias), or that the method is not able to estimate the respective confidence interval for the corresponding parameter combination (MCIL and FCR, see Materials and Methods).

We find that increasing the number of labels, *L*, continuously improves the estimation quality for both the activation and the proliferation rate (Fig 5). The absolute bias, as well as the false coverage rate, i.e. the probability that the actual rate is not within the calculated confidence interval, is reduced. Increasing the number of cells per label, *M*, only improves the robustness of parameter estimates judged by a decreasing mean confidence interval length (MCIL). Here, we observe a sharp decline between a strategy using unique labels and those relying on multiple cells per label. However, this effect quickly saturates in our scenario with increasing label sizes.

In some cases, confidence intervals for the activation rate, *μ*, cannot be obtained and the MCIL cannot be calculated. This is indicated by grey colour in the plots. In these cases, all activation rates above a certain threshold are equally likely to generate the observed outcome, leading to unlimited confidence intervals. This effect is mostly limited to labelling strategies with a low number of labels, *L*, but is also observed for unique labelling approaches having an intermediate number of labels (Fig 5C).

In summary, these results argue for the use of a large number of labels with medium numbers of cells per label as a reliable and robust labelling strategy.

#### Influence of the distribution of labels

Our previous analyses indicate that a large number of different labels reduces estimation bias while the use of larger label sizes generally improves the robustness of parameter estimates. As only a limited number of cells can be transferred, this leads to the question if estimation can be improved by a combination of both approaches. For example, does a strategy relying on many labels with small label sizes and few labels with more cells per label perform better than one using unique labels for all cells?

**Fig 6:**
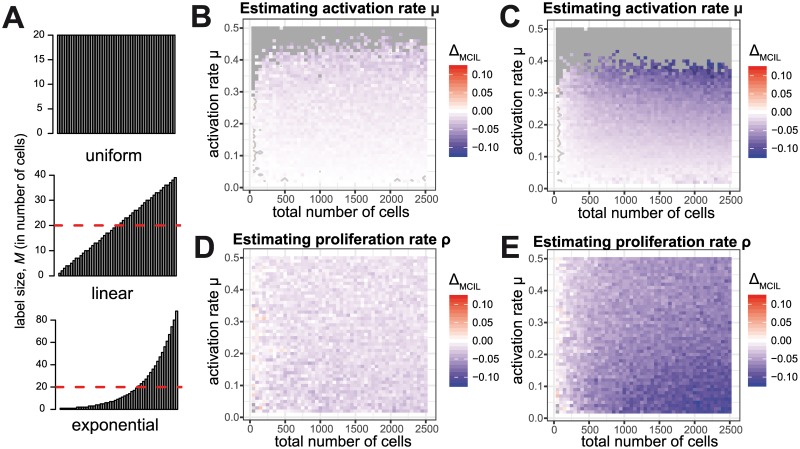
Influence of the label size distribution on parameter estimation. (**A**) Examples of the three different distributions of label sizes investigated: Uniform, linear and exponential distribution (from top to bottom). Each distribution comprises a total of 1000 cells and 50 labels. The red dotted line indicates the average label size of 20 cells. (**B-C**) The difference in the MCIL for the estimation of the activation rate *μ* between the uniformly and linearly distributed labels (**B**), and the difference between uniformly and exponentially distributed labels (**C**). (**D**, **E**) Analogous to (**B**, **C**) the difference in the MCIL for the estimated proliferation rate *ρ*.

To address this question, we repeated our analyses by using a fixed total number of cells that were labelled with *L* different markers using either a uniformly, linearly or exponentially distributed label size (Fig 6A). The evaluation of data from non-uniformly distributed label sizes required the adaptation of our approach for the calculation of the corresponding summary statistics (S1 Appendix). To compare the performance of the different labelling strategies, we then calculated the difference between the bias, the FCR, and the mean-confidence interval length (e.g. Δ_*MCIL*_ = MCIL_**Uniform**_ − MCIL_**Linear**_).

We find that a uniformly distributed labelling strategy always performs best in terms of estimation bias and robustness of parameter estimates independent of the total number of cells transferred (Fig 6B–6E for Δ_MCIL_, plots for pBias and FCR not shown). This observation is consistent for the activation, *μ*, and proliferation rate *ρ*. Increasing the inequality between label sizes impairs the quality of parameter estimates as an exponentially distributed labelling strategy always performs worst. Thus, a combination of several uniquely labelled cells with few labels comprising multiple cells does not improve parameter identification compared to an approach based on the same number of labels uniformly distributed among the cells.

#### The influence of the sampling time

In our scenario, we investigate the impact of various labelling strategies on inferring cellular dynamics if measurements can only be obtained at a single time point. Thereby, the choice of this sampling time point, *T*, also has an influence on the ability to estimate the kinetics.

**Fig 7:**
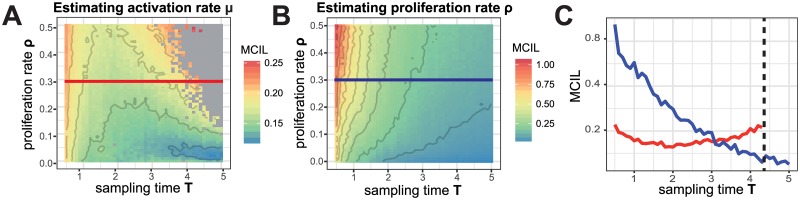
Influence of the sampling time on parameter estimation. MCIL of the activation rate, *μ* (**A**), and proliferation rate, *ρ* (**B**), using varying combinations of sampling times, *T*, and proliferation rates, *ρ*, in the simple system of cell activation and proliferation. Panel (**C**) shows the cross sections of panels (**A** & **B**) indicating the MCIL of the activation rate, *μ*, (*red*) and the proliferation rate, *ρ*, (*blue*) dependent on the sampling time for a fixed proliferation rate (*ρ* = 0.3). The black dotted line defines the time after which the estimation of the activation rate failed as all labels were sampled. The estimation for each parameter combination is based on 100 independent stochastic simulations. Parameters that were kept fixed are *μ* = 0.3, *L* = 50 and *M* = 5. Grey colour indicates that the method is not able to estimate the respective confidence interval for the corresponding parameter combination (see Materials and Methods).

For example, if in our scenario of cell activation and subsequent proliferation the sampling time point is chosen too late, the activation rate *μ* cannot be reliably estimated (Fig 7A). In contrast, sampling too early leads to increased uncertainty in the estimates due to stochastic effects. Thus, sampling at an intermediate time point gives the most reliable estimates for the activation rate. In contrast, for the proliferation rate *ρ* we observe that a later sampling time continuously improves robustness of the estimates (Fig 7B, MCIL) and parameter identification, i.e. leading to a reduced percentage bias (S4 Fig). Thus, there is a trade-off regarding the time point of sampling leading to more certainty in the estimates for either the activation or the proliferation rate (Fig 7C).

This trade-off is also found in the complex expansion system (see S1 Fig). Here, proliferation rates are estimated more reliably as time increases, while the transition from naïve to central memory precursor cells is captured especially well for early sampling time points. In case of the homoeostatic system, no effect of the sampling time on the parameter estimation is observed, and both the proliferation and death rate are estimated reliably irrespective of the sampling time (S1 Fig).

In summary, our results show that the identification of proliferation rates benefits from later sampling times, while initially occurring transition dynamics might already be masked by then. Hence, an appropriate estimation of all involved dynamics might not be possible in many systems.

## Discussion

Over the last decades, technical advances have steadily increased the possibilities to label cells by specific markers. As of today, a huge variety of labelling methods in various levels of detail exists, relying on naturally occurring or artificially induced cellular markers. These methods have been applied in adoptive transfer experiments to quantify cellular differentiation and expansion dynamics (reviewed in [6, 21]). However, to which extent the various labelling strategies actually allow the appropriate identification and quantification of the processes characterising the cellular dynamics has not been systematically studied.

To address this question, we simulated adoptive transfer experiments with various labelling strategies for different scenarios of cellular turnover. These scenarios included homoeostatic cell proliferation, as well as simple and complex expansion dynamics involving several steps of cell differentiation. We particularly focused on the situation where only one single measurement can be obtained, as e.g. due to organ harvest [2, 16].

In general, we found that a larger number of labels continuously improves parameter estimation in all of the different models tested. This is not completely surprising, as each label provides an additional measurement that can be used in the analysis and, thereby, reduces estimation bias and variance.

Testing two extreme labelling strategies involving either the transfer of 800 uniquely labelled cells [2] or using only 8 labels with 100 cells each [16], we found that the complexity of the system influences the required number of labels. Both labelling strategies showed a similar average bias for the quantification of cell proliferation and death within a homoeostatic model, with the unique labelling approach leading to less variation (Figs 2 and 4). However, within a system of complex cell expansion and differentiation dynamics as considered by Buchholz et al. [16], a shared labelling approach similar to the one used in their experiment generally leads to a slight systematic bias when estimating cell differentiation and proliferation rates. Only rates associated with intermediate compartments for which measurements of the previous and subsequent differentiation steps can be obtained (i.e. EM compared to CM and E, as N was not measured) can be reliably identified (Figs 3 and 4). In addition, the relative relationship between the cell proliferation and expansion rates of the different compartments could not be recovered in the estimates. This suggests that previous estimates for the proliferation and differentiation dynamics of T cells [16] should be taken with care as the labelling approach might be insufficient to determine those rates reliably.

Unique labelling is usually preferred to infer lineage differentiation pathways, such as for immune cell differentiation [1, 2, 16, 26] or hematopoiesis [34–36]. However, our analysis indicates that unique labelling is not always the best approach when estimating cellular turnover or expansion. Estimates on the proliferation dynamics are more robust if larger label sizes are used. Such label sizes will make the labelled population less prone to stochastic effects, although this improvement quickly saturates with increasing label sizes—at least for the analysed simple expansion dynamics.

Interestingly, we found that strategies combining labels with smaller and larger label sizes perform worse than uniformly distributed labels. In general, a strategy using uniformly distributed label sizes provided the most reliable results. However, an approach combining unique and large labels can still be beneficial, as the unique labels can be used to estimate the potential fraction of cells lost during transfer [16]. Due to the varying dependency of cell differentiation and proliferation rates on population sizes, a trade-off can be observed with regard to the choice of the sampling time. Proliferation rates belonging to continuously expanding cell compartments are estimated more reliably given later sampling times. However, harvesting cells at a late time point might severely impair the estimation of activation or initial transition dynamics. Hence, a robust estimation for all parameters might not be achievable if only one sampling time point is available.

The possible loss of cells during adoptive transfer or by incomplete sampling are experimental limitations that can strongly impact the quantification of cellular dynamics. Prior-knowledge on these quantities could be used to correct parameter estimation. However, while transfer loss could be experimentally approximated by using unique labels [16], determining the actual fraction of cells sampled remains difficult. Experimental methods might bias measurements against certain cellular subsets and thereby underestimate the total cell population [32]. However, sampling only a fraction of cells particularly affected cellular expansion rates while transition rates remained mostly identifiable.

In our analyses, we generally assumed that each label is independent and stable. Furthermore, we assumed that the label itself does not interfere with the underlying cellular dynamics. While this is appropriate for artificial markers, such as genetic barcodes [2] or unrelated congenic markers [4, 16], this assumption will most likely be violated in case of naturally occurring markers, such as *α*− and *β*-chains of T cell receptors (TCR) [20, 37, 38]. Here, the actual *β*-chain could affect T cell affinity and, thus, influences T cell activation [39, 40]. In addition, due to TCR *β*-chain rearrangements, these labels might not be considered as stable, impairing the possibility to track populations of cells [41, 42]. Novel analysis methods have to be developed to determine if such markers can still be used to infer cellular dynamics.

In summary, our results suggest that a generally suitable labelling strategy consists of a large number of shared labels, with an intermediate number of cells per label. This approach would likely lead to reliable estimates for different cellular systems, even in the case of incomplete transfer or sampling. In general, assumed model systems should always be tested in the context of the applied experimental labelling strategies in order to validate obtained parameter estimates. Performing a-priori simulations or a-posteriori testing allows to identify potential pitfalls, such as consistent bias or a susceptibility of parameter estimates to incomplete transfer or sampling. More systematic analyses of the relationship between labelling strategies and specific cellular systems are needed to infer appropriate labelling strategies in terms of actual cell numbers. Advances in single cell technologies [43, 44] and cell sorting might provide the necessary techniques to customise labelled cell populations used for adoptive transfer.

## Supporting information

S1 Appendix Mathematical derivation of estimation methods. (PDF)

S1 Fig Parameter estimates for the homoeostatic and complex expansion system given different sampling times. (PDF)

S2 Fig Parameter estimates for the complex expansion system corrected by the transfer fraction. (PDF)

S3 Fig Parameter estimates for the homoeostatic and complex expansion system corrected by the sampling fraction. (PDF)

S4 Fig Influence of the sampling time on the estimation quality given the simple expansion model. (PDF)
