# Supplementary material for: More or less—On the influence of labelling strategies to infer cell population dynamics
Source: PLoS One. 2017 Oct 18;12(10):e0185523. doi: 10.1371/journal.pone.0185523 (PMC5646766; doi:10.1371/journal.pone.0185523)
Supplement: S1 Appendix — (PDF) [file pone.0185523.s001.pdf]

Text S1: Details on the mathematical methods

We will show the derivation of the master equation and the fitting procedure based on the complex expansion model. Here, we also considered the death rates within the individual compartments. The other two scenarios (homoeostatic turnover and simple expansion dynamics) can easily be derived from this model by setting certain parameters equal to zero.

S1.1 - Solving the master equations

We distinguish between naïve (N), central memory precursor (CM), effector memory precursor (EM) and effector cells (E). The relation between these compartments is given by the following linear differentiation pathway:

$$\begin{aligned} \frac{dN}{dt} &= -\mu_N N \\ \frac{dCM}{dt} &= \mu_N N + (\rho_{CM} - \delta_{CM} - \mu_{CM})CM \\ \frac{dEM}{dt} &= \mu_{CM} CM + (\rho_{EM} - \delta_{EM} - \mu_{EM})EM \\ \frac{dE}{dt} &= \mu_{EM} EM + (\rho_E - \delta_E)E \ , \end{aligned} \tag{1}$$

The system given in Eq. (1) can also be formulated stochastically by a continuous-time Markov process which is described by the master equations for the state probabilities $p_{k,l,m,n}(t)$ derived from the corresponding transition probabilities. The master equation for the state probability $p_{k,l,m,n}(t)$, which describes the probability that we observe N= k, CM= l, EM= m and E= n cells in the different compartments at time t, is then determined by

$$\begin{aligned} \frac{dp_{k,l,m,n}}{dt} &= \mu_N(k+1)p_{k+1,l-1,m,n} + \mu_{CM}(l+1)p_{k,l+1,m-1,n} + \mu_{EM}(m+1)p_{k,l,m+1,n-1} \\ &+ \rho_{CM}(l-1)p_{k,l-1,m,n} + \rho_{EM}(m-1)p_{k,l,m-1,n} + \rho_E(n-1)p_{k,l,m,n-1} \\ &+ \delta_{CM}(l+1)p_{k,l+1,m,n} + \delta_{EM}(m+1)p_{k,l,m+1,n} + \delta_E(n+1)p_{k,l,m,n+1} \\ &- (\mu_N k + (\rho_{CM} + \delta_{CM} + \mu_{CM})l + (\rho_{EM} + \delta_{EM} + \mu_{EM})m + (\rho_E + \delta_E)n)\, p_{k,l} \, . \end{aligned} \tag{2}$$

We can derive the probability generating function that is defined as

$$F(z_0, z_1, z_2, z_3) = \sum_{(n_0,n_1,n_2,n_3)} z_0^{n_0} z_1^{n_1} z_2^{n_2} z_3^{n_3} P(n_0, n_1, n_2, n_3; t) \tag{3}$$

with the state vector (n_0, n_1, n_2, n_3) characterising the populations of naïve (n_0), central memory precursor (n_1), effector memory precursor (n_2) and effector cell (n_3) at time point t. Inserting Eq. (3) in the master equation Eq. (2) leads to the following partial differential equation:

$$\partial_t F = \sum_{i=1}^{3} \mu_i (z_i - z_{i-1}) \partial_{z_{i-1}} F + \delta_i (1 - z_i) \partial_{z_i} F + \rho_i (z_i^2 - z_i) \partial_{z_i} F \tag{4}$$

On the other hand, the probability generating function can be derived with regard to z_i and evaluated at $z_i = 1,\ i = 1, ..., 4$ which gives the first factorial moments

$$\frac{dF}{dz_i}|_1 = \sum_{(n_0,n_1,n_2,n_3)} n_i P(n_0, n_1, n_2, n_2; t) \tag{5}$$

The second derivative yields the second moments, and so on. By defining $x_N := \partial z_0 F|_{z_i=1}$, $y_N := \partial z_0^2 F|_{z_i=1}$ and $c_{N,CM} := \partial z_0 z_1 F|_{z_i=1}$, ..., we can split the partial differential equation in Eq. (4) into a system of ordinary differential equations:

$$
\begin{aligned}
\frac{dx_N}{dt} &= -\mu_{CM} x_N \\
\frac{dx_{CM}}{dt} &= \mu_{CM} x_N + (-\mu_{EM} - \delta_{CM} + \rho_{CM}) x_{CM} \\
\frac{dx_{EM}}{dt} &= \mu_{EM} x_{CM} + (-\mu_E - \delta_{EM} + \rho_{EM}) x_{EM} \\
\frac{dx_E}{dt} &= \mu_E x_{EM} + (-\delta_E + \rho_E) x_E
\end{aligned}
$$

$$
\begin{aligned}
\frac{dy_{CM}}{dt} &= -\mu_{CM} y_N \\
\frac{dy_{CM}}{dt} &= \mu_{CM} c_{N,CM} + \rho_{CM} x_{CM} + (-\mu_{EM} - \delta_{CM} + \rho_{CM}) y_{CM} \\
\frac{dy_{EM}}{dt} &= \mu_{EM} c_{CM,EM} + \rho_{EM} x_{EM} + (-\mu_E - \delta_{EM} + \rho_{EM}) y_{EM} \\
\frac{dy_E}{dt} &= \mu_E c_{EM,E} + \rho_E x_E + (-\delta_E + \rho_E) y_E
\end{aligned} \tag{6}
$$

$$
\begin{aligned}
\frac{dc_{N,CM}}{dt} &= \mu_{CM} y_N + (-\mu_{CM} - \mu_{EM} - \delta_{CM} + \rho_{CM}) c_{N,CM} \\
\frac{dc_{N,EM}}{dt} &= \mu_{EM} c_{N,CM} + (-\mu_{CM} - \mu_E - \delta_{EM} + \rho_{EM}) c_{N,EM} \\
\frac{dc_{N,E}}{dt} &= \mu_E c_{N,EM} + (-\mu_{CM} - \delta_{CM} + \rho_{CM}) c_{N,E} \\
\frac{dc_{CM,EM}}{dt} &= \mu_{CM} c_{N,EM} + \mu_{EM} y_{CM} + (-\mu_{EM} - \mu_E - \delta_{CM} - \delta_{EM} + \rho_{CM} + \rho_{EM}) c_{CM,EM} \\
\frac{dc_{CM,E}}{dt} &= \mu_{CM} c_{N,E} + \mu_E c_{CM,EM} + (-\mu_{EM} - \delta_{CM} - \delta_E + \rho_{CM} + \rho_E) c_{CM,E} \\
\frac{dc_{EM,E}}{dt} &= \mu_{EM} c_{CM,E} + \mu_E y_{EM} + (-\mu_E - \delta_{EM} - \delta_E + \rho_{EM} + \rho_E) c_{EM,E} \ .
\end{aligned}
$$

The solutions to these equations can be used to calculate mean, variance and covariance of the different cell populations. It holds that:

$$
\begin{aligned}
\mathbb{E}[N] &= x_N(t) \\
\mathbf{Var}[N] &= y_N(t) + x_N(t) - x_N(t)^2 \\
\mathbf{Cov}[N, CM] &= c_{N,CM}(t) - x_N(t) x_C M(t) \ .
\end{aligned} \tag{7}
$$

The remaining statistics are defined accordingly.

The summary statistics used for fitting are the expected mean, the coefficients of variation (CV) and the correlation coefficients (CC). The latter two are calculated by

$$\begin{aligned} \mathbf{CV}[i] &= \frac{\sqrt{\mathbf{Var}[i]}}{\mathbb{E}[i]} \\ \mathbf{CC}[i,j] &= \frac{\mathbf{Cov}[i,j]}{\sqrt{\mathbf{Var}[i]\mathbf{Var}[j]}} \;. \end{aligned} \tag{8}$$

S1.2 - Adjusting for transfer loss

Losing cells during adoptive transfer can be considered as taking a sample from the transferred cell population that defines the effective initial population of cells. The number of cells that each label contributes to the transferred labeling strategy can be calculated by using the hypergeometric distribution, which needs three input variables: the number of cells in the chosen label, M, the overall number of cells, N and the considered fraction of cells, p, that will survive the transfer. While M and N are known from the initial labelled population, the transfer fraction p needs to be experimentally determined. If all values are known, we can calculate the expected number and variance of the transferred cells by

$$\begin{aligned} \mathbb{E}(\#\text{ of transferred cells}) &= pM \\ \mathbf{Var}(\#\text{ of transferred cells}) &= p(1-p)\frac{M(N-M)}{N-1} \;. \end{aligned} \tag{9}$$

Using Eqs. (7), these values can be used as initial conditions for the system in Eq. (6).

S1.3 - Deriving estimates from non-uniformly distributed initial conditions

If the initial labeling strategy is not uniformly distributed, the obtained output data need to be adjusted to derive the summary statistics. The idea is to rescale all values to achieve comparability. This is possible because the calculated mean and variance of Eq. 7 scale linearly with the initial number of transferred cells, if and only if all other cell compartments are empty at time $t = 0$. For our models this condition is fulfilled since we assumed no activated cells exist in the beginning.

To be more exact, if X_M describes the number of further differentiated cells at sampling time T, derived from a initial population of M equally labelled cells, it holds that:

$$\mathbb{E}(X_M) = M\mathbb{E}(X_1) \tag{10}$$

$$\mathbf{Var}(X_M) = M\mathbf{Var}(X_1) \ . \tag{11}$$

This means the distribution of shared labels at time T can be derived from the calculated quantities based on a unique label.

Now considering a non-uniformly distributed labeling strategy made up of L different labels, we denote M_i as the initial number of cells of label i and X_i as the corresponding sampled cell population at time T. We define the quantities

$$E_1 := \frac{\sum_{i=1}^{L} X_i}{\sum_{i=1}^{L} M_i} \tag{12}$$

$$V_1 := \sum_{i=1}^{L} (X_i - M_i E_1)^2 \ . \tag{13}$$

$\mathbb{E}_1$ is the expected value of a population starting with $M = 1$ cell. However, for V_1 we see that

$$\begin{aligned}
\mathbb{E}[V_1] &= \sum_{i=1}^{L} \mathbb{E}[X_i - M_i E_1[^2 = \sum_{i=1}^{L} \mathbb{E}[X_i - \mathbb{E}[X_i] + \mathbb{E}[X_i] - M_i E_1]^2 \\
&= \sum_{i=1}^{L} \mathbb{E}[(X_i - \mathbb{E}[X_i])^2 + 2(X_i - \mathbb{E}[X_i])(\mathbb{E}[X_i] - M_i E_1) + (\mathbb{E}[X_i] - M_i E_1)]^2 \\
&= \sum_{i=1}^{L} \mathrm{Var}[X_i] + \sum_{i=1}^{L} \mathbb{E}[2(X_i - \mathbb{E}[X_i])(\mathbb{E}[X_i] - M_i E_1)] + \sum_{i=1}^{L} \mathrm{Var}[M_i E_1] \\
&= M\mathrm{Var}[X_1] - \frac{2}{M} \sum_{i=1}^{L} \mathbb{E}\left[(X_i - \mathbb{E}[X_i])(M_i \sum_{j=1}^{L} X_j - M\mathbb{E}[X_i]) \right] \\
&\quad + \frac{(\sum_{i=1}^{L} M_i^2)}{M} \mathrm{Var}[X_1] \\
&= M\mathrm{Var}[X_1] - \frac{2}{M} \sum_{i=1}^{L} \mathbb{E}\left[(X_i - \mathbb{E}[X_i])(\sum_{j=1}^{L} M_i (X_j - \mathbb{E}[X_i])) \right] \\
&\quad + \frac{(\sum_{i=1}^{L} M_i^2)}{M} \mathrm{Var}[X_1] \\
&= M\mathrm{Var}[X_1] - \frac{2}{M} \sum_{i=1}^{L} M_i \mathbb{E}[(X_i - \mathbb{E}[X_i])^2] + \frac{(\sum_{i=1}^{L} M_i^2)}{M} \mathrm{Var}[X_1] \\
&= M\mathrm{Var}[X_1] - \frac{2\sum_{i=1}^{L} M_i^2}{M} \mathrm{Var}[X_1] + \frac{(\sum_{i=1}^{L} M_i^2)}{M} \mathrm{Var}[X_1] \\
&= \frac{M^2 - \sum_{i=1}^{L} M_i^2}{M} \mathrm{Var}(X_1) \ .
\end{aligned}$$

Hence, correcting $V1$ by $\frac{M}{M^2 - \sum_{i=1}^{L} M_i^2}$ leads to an unbiased estimator of the variance of X_1, which can be used in the fitting procedure.
