# Supplementary material for: More or less—On the influence of labelling strategies to infer cell population dynamics
Source: PLoS One. 2017 Oct 18;12(10):e0185523. doi: 10.1371/journal.pone.0185523 (PMC5646766; doi:10.1371/journal.pone.0185523)
Supplement: S1 Fig — (PDF) [file pone.0185523.s002.pdf]

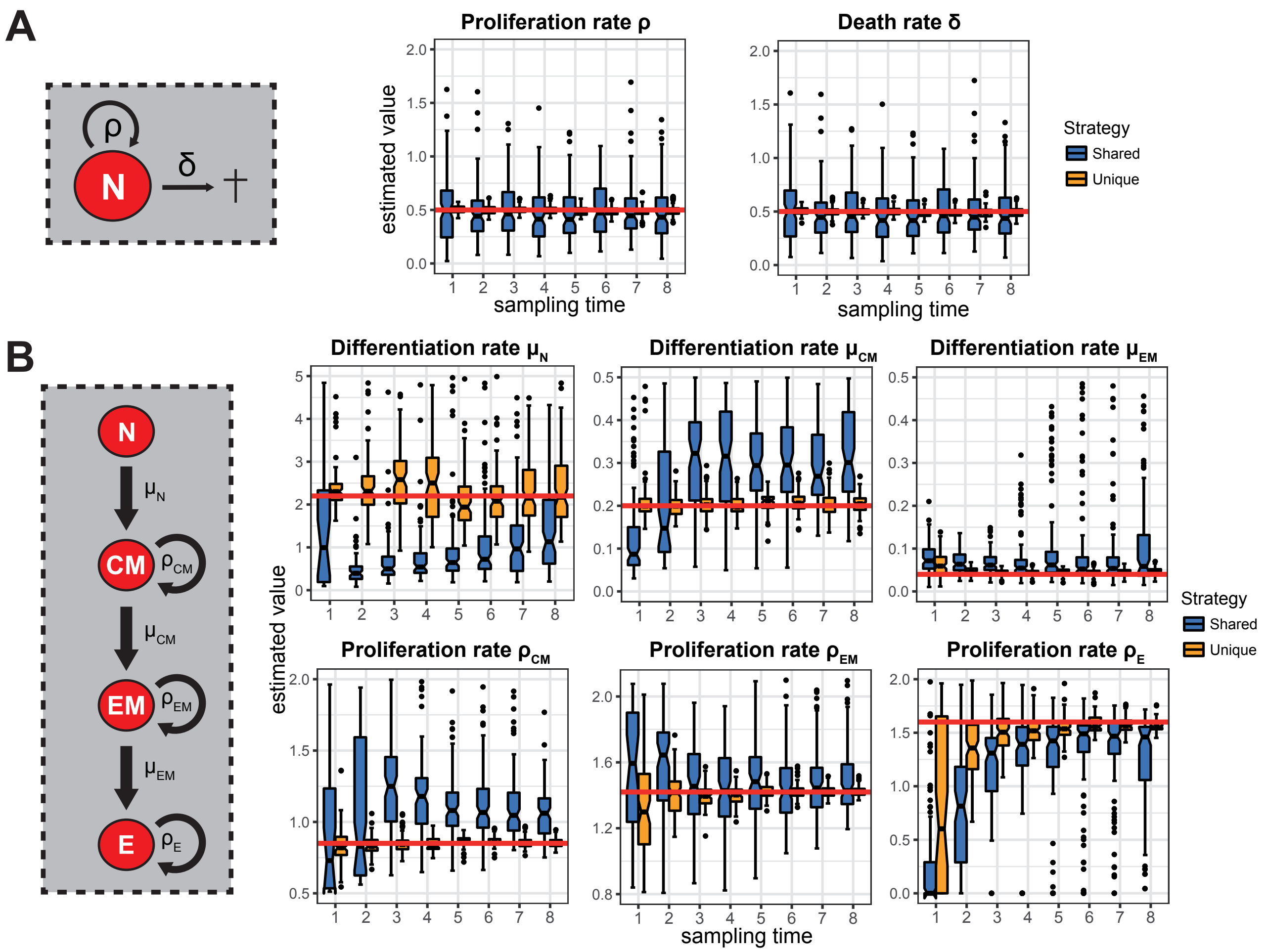

Fig S1: Parameter estimates for the homoeostatic and complex expansion system given different sampling times: (**A**) Estimation of proliferation and death rates given the depicted homoeostatic system. (**B**) The complex expansion system with corresponding differentiation and proliferation rates. Panels show the estimated rates given different sampling times using a shared ($L = 8,\ M = 100,$ *blue*) and a unique ($L = 800,\ M = 1,$ *orange*) labelling strategy. Here, no loss of cells during transfer or sampling was assumed. Every boxplot is based on the results of 100 independent stochastic simulations. Red lines indicate the true parameter values.
