# Supplementary material for: More or less—On the influence of labelling strategies to infer cell population dynamics
Source: PLoS One. 2017 Oct 18;12(10):e0185523. doi: 10.1371/journal.pone.0185523 (PMC5646766; doi:10.1371/journal.pone.0185523)
Supplement: S2 Fig — (PDF) [file pone.0185523.s003.pdf]

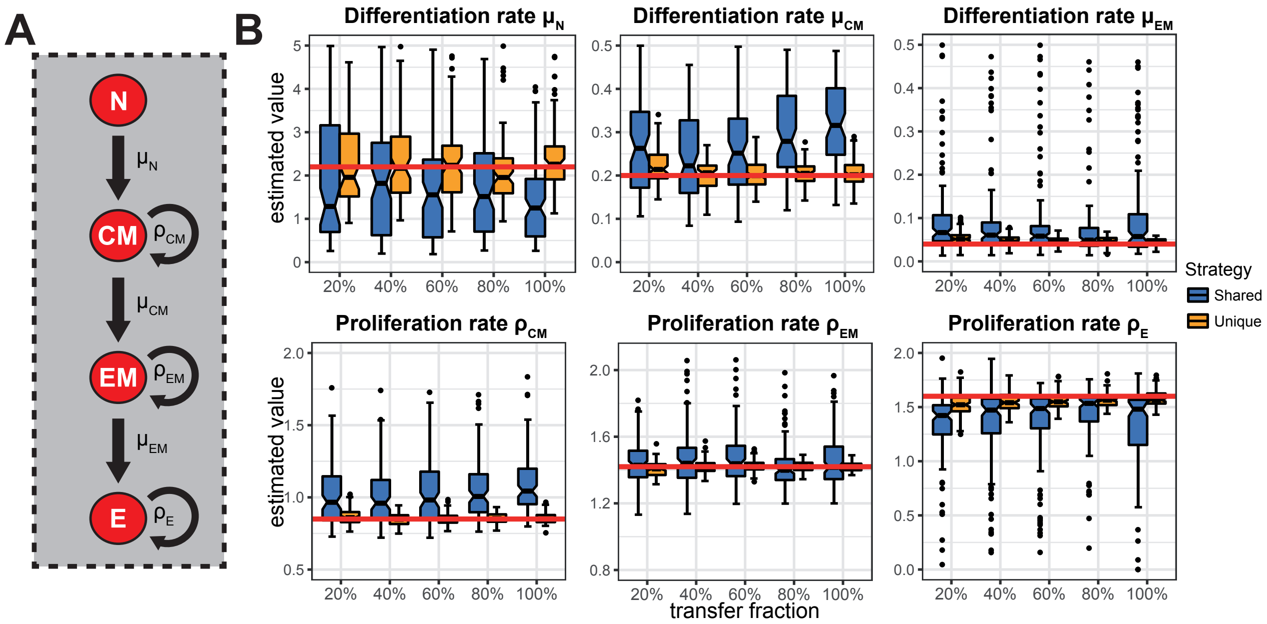

Fig S2: Parameter estimates for the complex expansion system corrected by the pre-known transfer fraction: (**A**) The complex expansion system with corresponding differentiation and proliferation rates. (**B**) Panels show the estimated rates given different transfer fractions using a shared ($L = 8,\ M = 100,$ *blue*) and a unique ($L = 800,\ M = 1,$ *orange*) labelling strategy. Here, we accounted for the loss in transfer. Every boxplot is based on the results of 100 independent stochastic simulations. Red lines indicate the true parameter values.
