# Supplementary material for: More or less—On the influence of labelling strategies to infer cell population dynamics
Source: PLoS One. 2017 Oct 18;12(10):e0185523. doi: 10.1371/journal.pone.0185523 (PMC5646766; doi:10.1371/journal.pone.0185523)
Supplement: S3 Fig — (PDF) [file pone.0185523.s004.pdf]

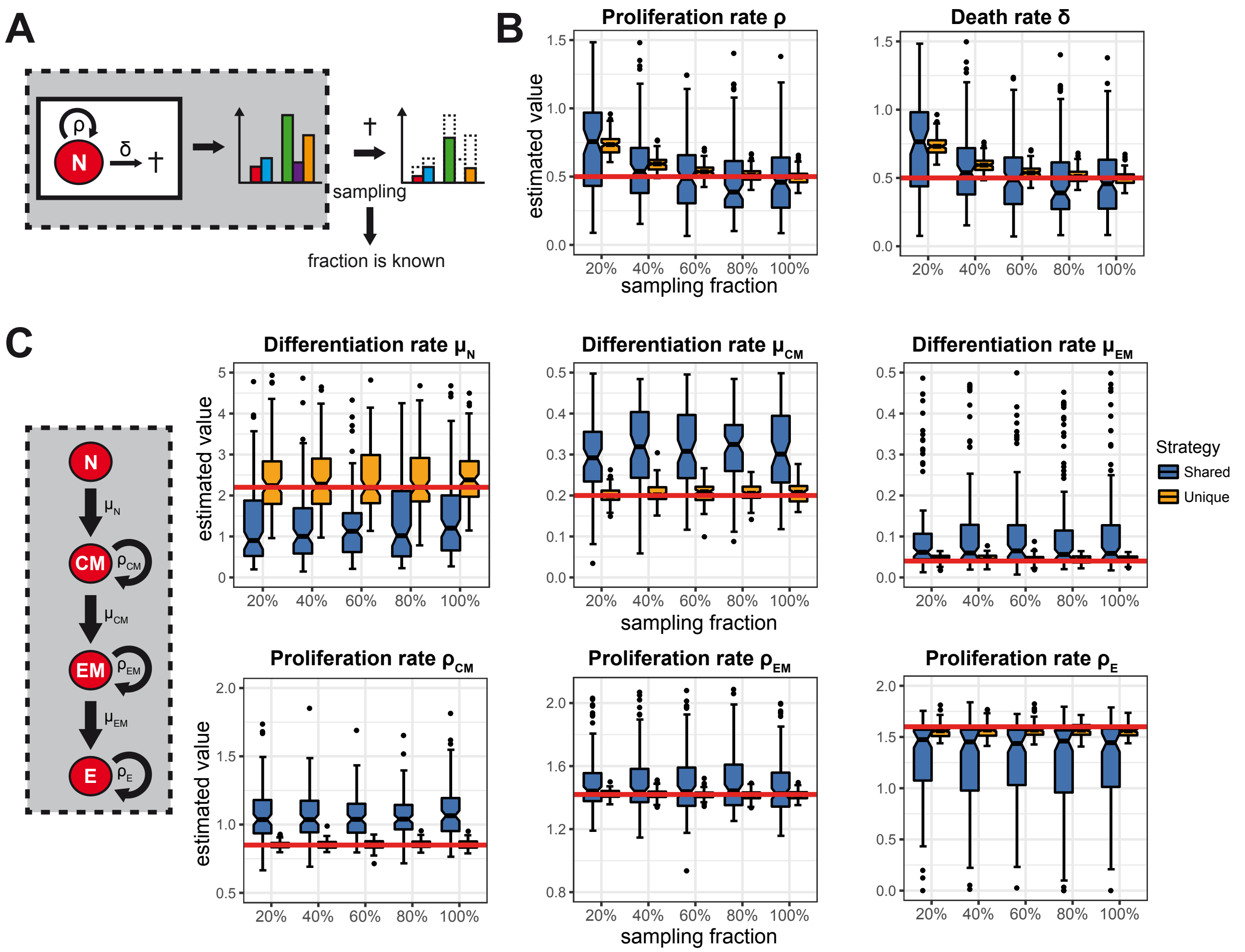

Fig S3: Parameter estimates for the homoeostatic and the complex expansion system corrected by the pre-known sampling fraction: (**A**) Schematic depicting the problem of incomplete sampling: Only a fraction of the labelled cells is sampled and can be used for analysis. Here, the sampling fraction is known. (**B**) Estimates for proliferation and death rate given the homoeostatic system corrected by the sampling fraction. (**C**) The complex expansion system with corresponding differentiation and proliferation rates. Panels show the estimated rates given different sampling fractions using a shared ($L = 8,\ M = 100,$ *blue*) and a unique ($L = 800,\ M = 1,$ *orange*) labelling strategy. Here, we accounted for the loss of cells during sampling. Every boxplot is based on the results of 100 independent stochastic simulations. Red lines indicate the true parameter values.
