# Supplementary material for: More or less—On the influence of labelling strategies to infer cell population dynamics
Source: PLoS One. 2017 Oct 18;12(10):e0185523. doi: 10.1371/journal.pone.0185523 (PMC5646766; doi:10.1371/journal.pone.0185523)
Supplement: S4 Fig — (PDF) [file pone.0185523.s005.pdf]

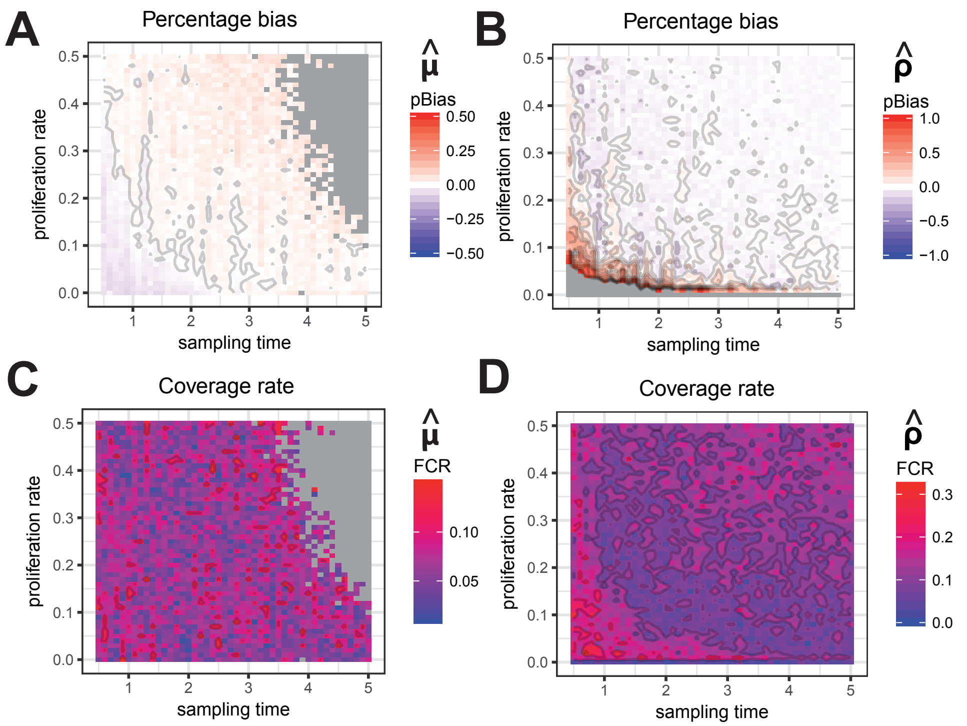

Fig S4: Influence of the sampling time on parameter estimates given the simple expansion model: (**A & B**) The percentage bias for the estimation of the activation rate, $\hat{\mu}$ (**A**), and the proliferation rate, $\hat{\rho}$ (**B**), based on varying combinations of sampling times, T, and proliferation rates, ρ. Grey areas in panel (**B**) indicate parameter combinations resulting in a percentage bias larger than 1. The corresponding false coverage rates are shown in panel (**C**) and (**D**), respectively. The estimated parameter for each panel is shown above the colour legend.
